# Autophagia in a Patient with Dementia and Hemineglect: A Case Report

**DOI:** 10.5811/cpcem.7228

**Published:** 2024-08-16

**Authors:** Bradley N. Bragg, Kara J. Bragg

**Affiliations:** Mayo Clinic, Department of Emergency Medicine, Jacksonville, Florida

**Keywords:** case report, patients living with dementia, self-injury, autophagia, hemineglect

## Abstract

**Introduction:**

Patients living with dementia as well as patients with neurological deficits are at significant risk for injury from multiple sources. Injuries may include falls, neglect, and, in some cases, self-injury. These patients require significant observation and closely monitored care.

**Case Report:**

A 90-year-old man presented to a suburban emergency department (ED) by his family, who cared for him at home. The following case report describes a patient with dementia, hemineglect, and bruxism from a previous stroke who suffered a self-induced, partial amputation of his own thumb on the neglected side of his body.

**Conclusion:**

Patients with dementia and neurologic deficits present frequently in the ED. These patients are at considerable risk of self-injury. The emergency physician should maintain vigilance in both screening for injuries and being aware of these risks when planning living arrangements after disposition from the ED.

## INTRODUCTION

Emergency departments (ED) frequently care for patients living with dementia (PLWD), and there is a significant need to develop care plans and research protocols for better management of this patient population.^2^ Patients living with dementia have additional risks for comorbidities associated with functional dependence, behavioral challenges, and psychological manifestations of this disease. 2 Patients with brain lesions or strokes can suffer from hemineglect, which shows a general lack of awareness of response to stimuli on the contralateral side from the lesion. 3 This case illustrates an example of personal neglect due to a form of hemi-inattention with a deficit of tactile sensation and pain, as well as proprioceptive awareness on the affected side of the body. 3 There is little research on PLWD with hemineglect and additional risk factors for comorbidities. Treatment requires a comprehensive approach to evaluation and discharge planning.

## CASE REPORT

A 90-year-old man presented to a suburban ED by his family, who cared for him at home. He had a history of dementia and right-sided neglect from a previous stroke four years prior. As a result of the previous stroke, he also had an oral fixation and a predisposition to grinding his teeth (bruxism). According to the history provided by family, the patient would intermittently grind his teeth, profoundly at times, but it appeared to worsen when he was sleeping. The patient reportedly fell asleep at 6:30 pm in the evening. His family checked on him at approximately 7:30 pm and found a large volume of blood on his bed with a partial amputation of his right thumb. Given the patient’s history of bruxism and dementia, the physician concluded that he inadvertently chewed through his own thumb.

Physical examination revealed multiple jagged lacerations surrounding the distal half of the right thumb, beginning at the proximal phalanx. The nail plate was disrupted with these lacerations. The proximal portion of the nail plate was removed from the nail fold. The most proximal portion of the proximal phalanx both palmar and dorsally had jagged lacerations such that the only thing attaching the distal thumb to the hand was a 1centimeter skin bridge on either side of the medial and lateral aspects of the thumb (Image).

The distal thumb appeared dusky in color and was cool to the touch. A pulse oximeter probe placed on the thumb did not register any oxygen saturation. The capillary refill in the thumb was sluggish, measuring over two seconds in duration. Due to the patient’s impaired mentation and hemineglect, sensation of the thumb was unable to be assessed.

A radiograph of the right thumb revealed a comminuted fracture through the base of the first proximal phalanx. There was mild radial subluxation with minimal displacement of the distal fracture fragment. Gas in the soft tissues surrounding the proximal phalangeal fracture was also noted adjacent to the laceration. No foreign bodies were visualized.

The physician consulted orthopedic surgery. The surgeon discussed options with the family with consideration to the cleanliness of the laceration, bony exposure, and poor viability of the distal digit, the orthopedic surgeon performed a complete amputation. The patient was given ampicillin-sulbactam intravenously in the ED for prophylaxis, and the procedure was performed by the orthopedic surgery resident at the bedside. The distal thumb was amputated followed by copious irrigation and closure of skin over the residual thumb. The patient was discharged from the ED with amoxicillin-clavulanate with follow-up in the orthopedic hand surgery clinic. The wound healed well without complication.

## DISCUSSION

Self-injurious behavior has been reported in the elderly on multiple occasions, making this patient population at particularly high risk, particularly when cared for at home by family members. 4, 5 Self-amputation of the hand and self-cannibalism have been reported in the past, customarily in the setting of psychiatric illness and not as the result of dementia. 6, 7 Schizophrenia and associated depersonalization or dissociation can account for this behavior. 8 Self-mutilation is commonly seen in clinical practice, although studies are limited and often associated with abuse or opioids. 8 In PLWD, the specific prevalence of self-induced injuries is not well documented, and most involve excoriations in the skin secondary to formication. 9 This case would indicate that the hemineglect from the stroke combined with dementia-induced behaviors put this patient at significant risk for autophagia. Injury precautions for patients at risk of self-injury should be strongly adhered to. Close observation and frequent physical assessments are indicated by caregivers. In Lesch-Nyhan patients with compulsive self-injury behavior, often related to biting, preventive mouthguards have been proposed as an option to prevent self-mutilation. 10 In this situation, restraint mitts may aid in prevention of self-mutilation of hands. 11

**CPC-EM Capsule**

What do we already know about this clinical entity?

*Little has been reported about patients living with dementia with hemineglect and risk factors for self-injury. While patients with dementia are recognized to be at increased risk for injury from trauma such as falls, cases of unintentional self-harm are rarely reported.*

What makes this presentation of disease reportable?

*This case demonstrates a rarely-reported incident of self-harm in a patient with dementia and hemineglect.*

What is the major learning point?

*Patients with dementia can present significant risk of self injury. Emergency physicians should keep these potential risks in mind when managing patients with dementia and hemineglect.*

How might this improve emergency medicine practice?

*This case provides an example of an injury that can result from patients with dementia where adequate precautions were not taken to prevent self-harm. Having insight about this risk may help guide decisions about patient care.*

## CONCLUSION

Recent trends in end-of-life care have resulted in the United States using more supportive care and has found that patients aged 65 years and older are more likely to die at home.^1^ With an increasing number of patients with dementia being cared for at home, this places an increased responsibility on the family and more support with caregiver support and palliative home-based care. 1 The emergency clinician should be aware of these issues and keep them in mind when arranging for home care of patients with dementia. The physician should maintain a high degree of vigilance to screen and prevent self-mutilation injuries in the ED given the substantial risk in this patient population. Additionally, noted risk of self-mutilation in a population with combined stroke deficits and dementia must be considered in discharge planning from the ED or hospital.

## Figures and Tables

**Image:**
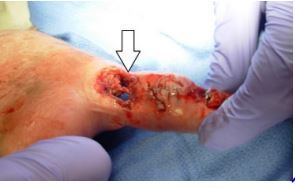
Thumb demonstrating self-mutilation of a patient with dementia, bruxism, and hemineglect.
